# Supplementary material for: Cultured meat with enriched organoleptic properties by regulating cell differentiation
Source: Nat Commun. 2024 Jan 2;15:77. doi: 10.1038/s41467-023-44359-9 (PMC10762223; doi:10.1038/s41467-023-44359-9)
Supplement: Supplementary file 1 — Supplementary Information [file 41467_2023_44359_MOESM1_ESM.pdf]

Supporting information

Cultured Meat with Enriched Organoleptic Properties by Regulating Cell Differentiation

Milae Lee[1], Sohyeon Park[1], Bumgyu Choi[1], Woojin Choi[1], Hyun Lee[2], Jeong Min Lee[3], Seung Tae Lee[2,3], Ki Hyun Yoo[4], Dongoh Han[4], Geul Bang[5], Heeyoun Hwang[5,6], Won-Gun Koh[1], Sangmin Lee[7], Jinkee Hong[1]

[1]Department of Chemical & Biomolecular Engineering, College of Engineering, Yonsei University, 50 Yonsei-ro, Seodaemun-gu, Seoul 03722, Republic of Korea

[2]Department of Animal Life Science, Kangwon National University, 1 Kangwondaehak-gil, Chuncheon-si, Gangwon-do 24341, Republic of Korea

[3]Department of Applied Animal Life Science, Kangwon National University, 1 Kangwondaehak-gil, Chuncheon-si, Gangwon-do 24341, Republic of Korea

[4]Simple Planet, 803, 34, sangwan 12-gil, Seongdong-gu, Seoul 04790, Republic of Korea

[5]Research Center for Convergence Analysis, Korea Basic Science Institute, Cheongju 29118, Republic of Korea

[6]Critical Diseases Diagnostics Convergence Research Center, Korea Research Institute of Bioscience and Biotechnology, Daejeon 34141, Republic of Korea

[7]School of Mechanical Engineering, Chung-ang University, 84, Heukseok-ro, Dongjak-gu, Seoul 06974, Republic of Korea

Correspondence is addressed to J.H. (email: jinkee.hong@yonsei.ac.kr) and S.L. (email: slee98@cau.ac.kr).

Figures

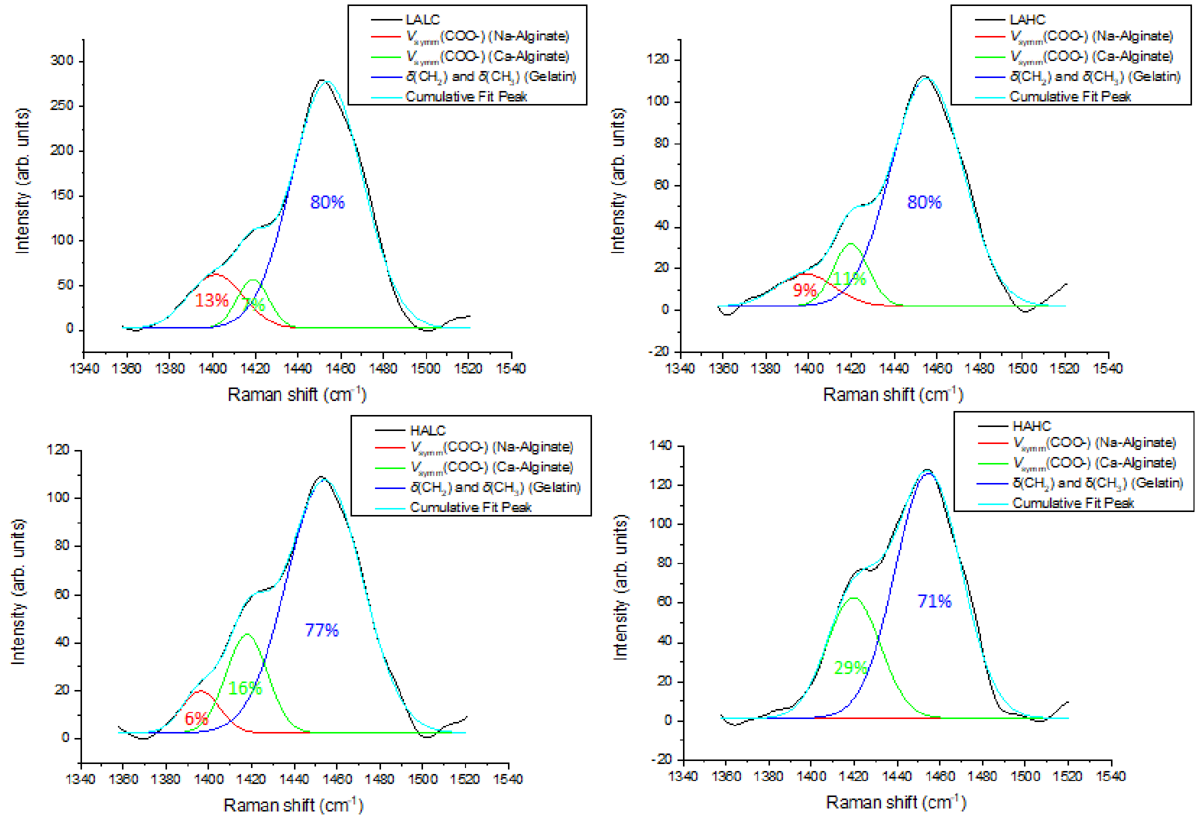

Supplementary Fig. 1: Raman spectra of the LALC, LAHC, HALC and HAHC, obtained in the Raman shift range from 1360 cm^{-1} to 1520 cm^{-1}. The area of the COO- peak assigned to sodium alginate and calcium alginate are shown. The CH_2 and CH_3 peaks represent gelatin.

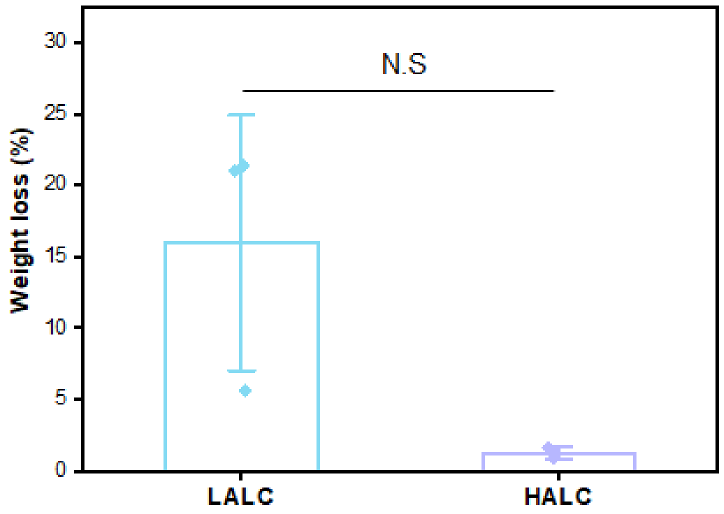

Supplementary Fig. 2: Degradation rates of the LALC and HALC. The degradation degree was quantified using the weight loss of the hydrogels (n = 3 independent experiments, two-tailed t-test). N.S indicates non-significant. Error bars represent mean ± SD. Source data are provided a Source Data file.

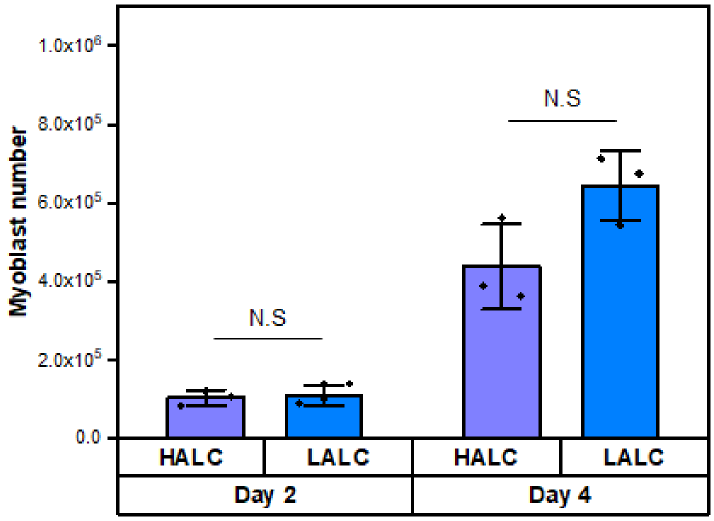

Supplementary Fig. 3: Evaluation of the cell proliferation of the bovine myoblasts. Viabilities of the bovine myoblasts of the culture well, HALC, and LALC group were compared on day 2 and day 4 (n = 3 independent experiments, two-tailed t-test). Error bars represent mean ± SD. Source data are provided a Source Data file.

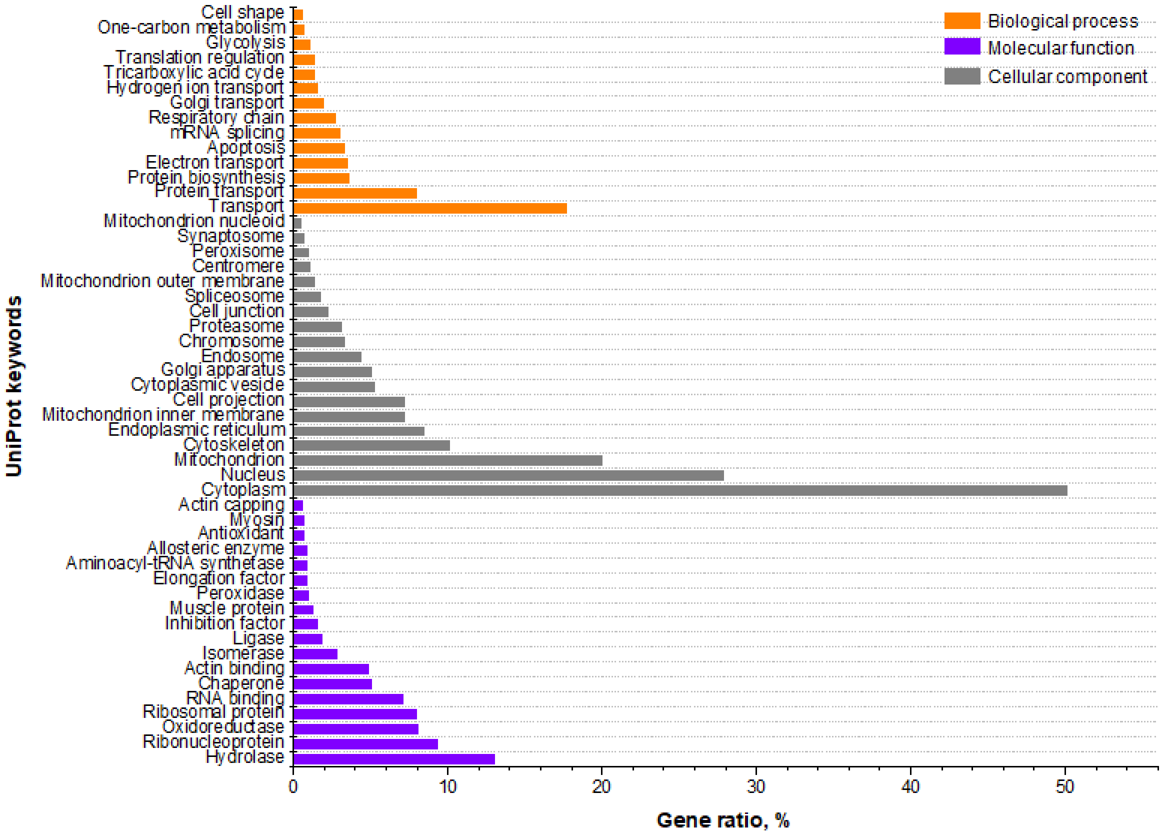

Supplementary Fig. 4: Gene annotation of the total up-regulated proteins in differentiated myoblasts of HALC. X-axis indicates the proportion of the genes with function corresponding to the gene annotation whereas Y-axis is the description of the gene functions. Source data are provided a Source Data file.

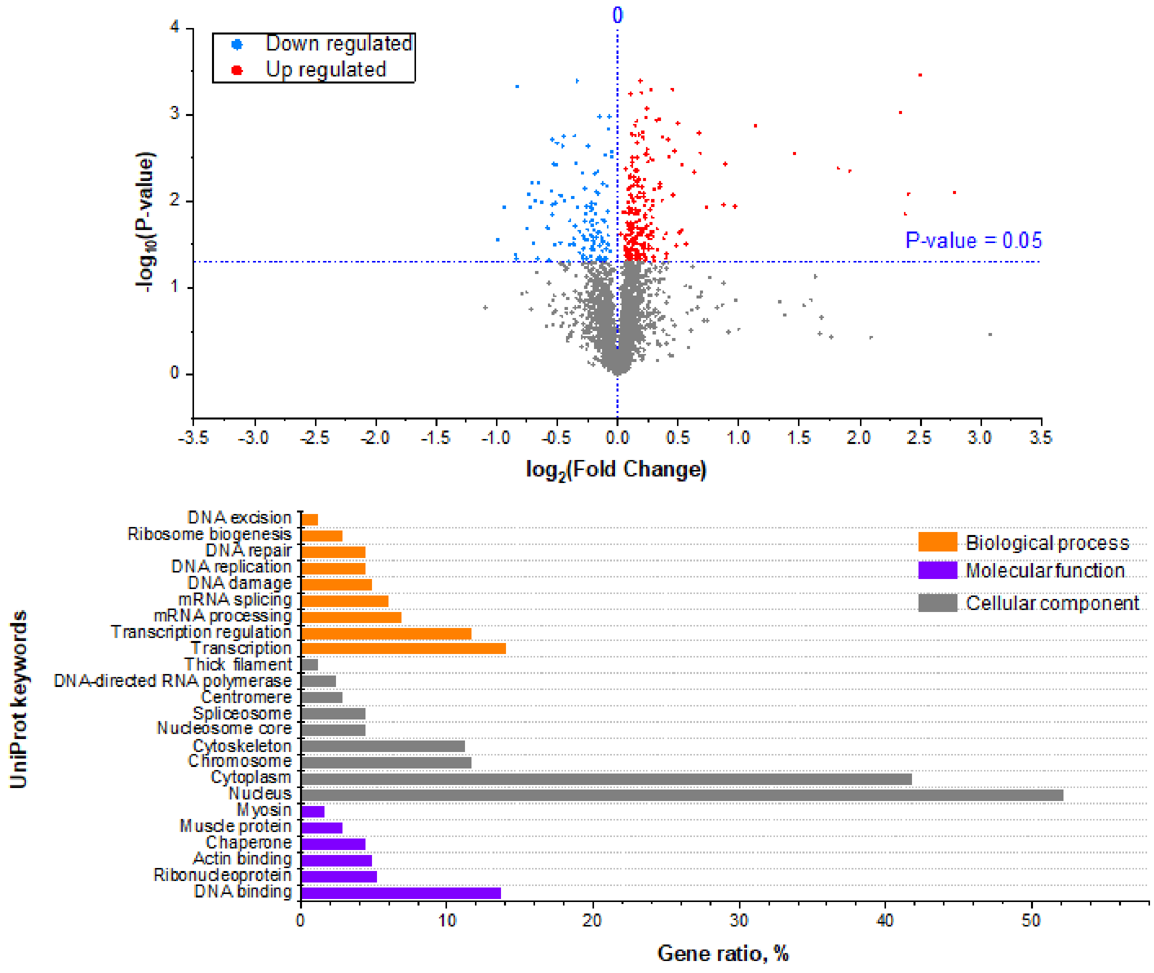

Supplementary Fig. 5: Proteins expressed in the undifferentiated myoblasts. Volcano plot indicates the significantly down-regulated (blue dots) and up-regulated (red dots) proteins in HALC compared to that of LALC (n = 3 independent experiments, two-tailed t-test). Fold change indicates the ratio of the protein expression intensity in HALC to the protein expression intensity in LALC. Gene annotation of the up-regulated proteins is shown below the volcano plot. X-axis indicates the proportion of the genes with function corresponding to the gene annotation whereas Y-axis is the description of the gene functions. Source data are provided a Source Data file.

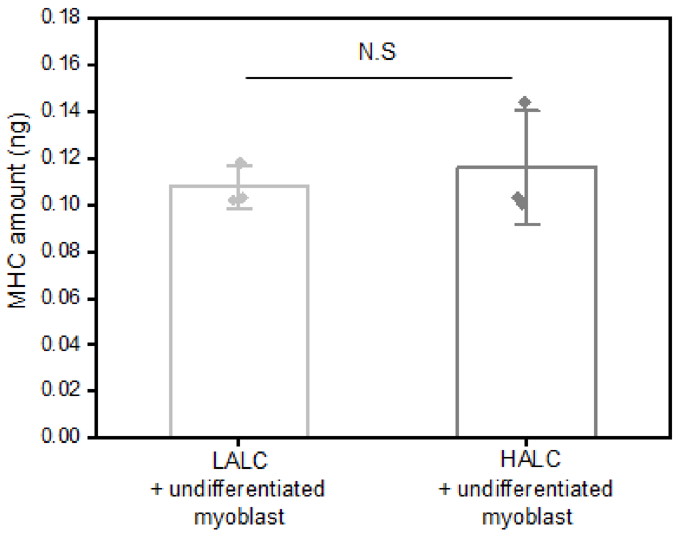

Supplementary Fig. 6: Amount of myosin heavy chain (MHC) expressed in the undifferentiated myoblasts in each scaffold. The expression of the myosin heavy chain in the undifferentiated samples was compared (n = 3 independent experiments, two-tailed t-test). N.S indicates non-significant. Error bars represent mean ± SD. Source data are provided a Source Data file.

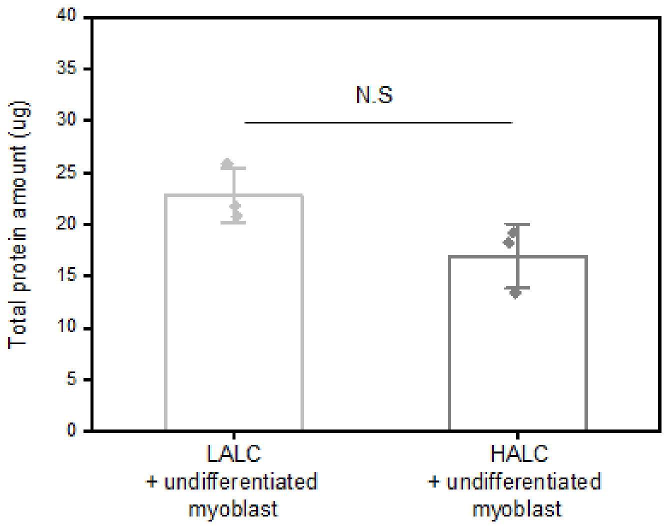

Supplemenary Fig. 7: Amount of total protein expressed in the undifferentiated myoblasts in each scaffold. Protein expression of the undifferentiated samples was evaluated using bicinchoninic acid (BCA) assay (*n* = 3 independent experiments, two-tailed t-test). N.S indicates non-significant. Error bars represent mean ± SD. Source data are provided a Source Data file.

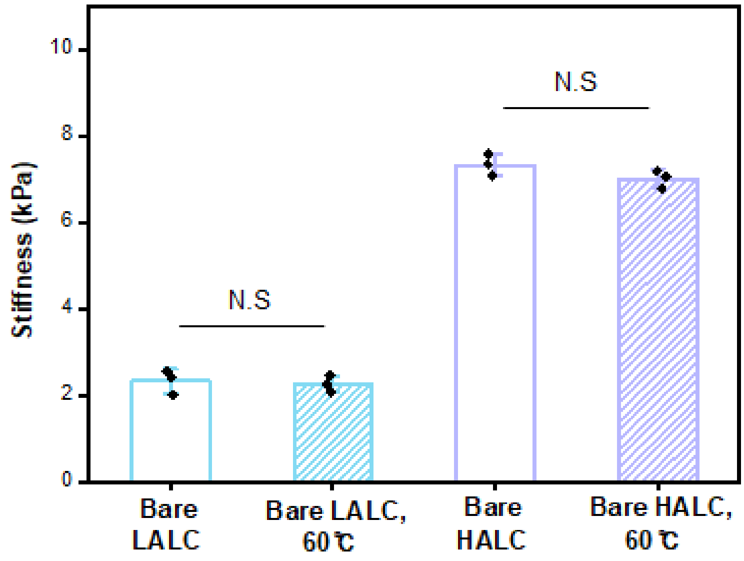

Supplemenary Fig. 8: Stiffness change of the bare scaffolds before and after cooking at 60 °C. The stiffness of each group was measured by frequency sweep at the angular frequency range from 0.1 to 1 (rad/s) (n = 3 independent experiments, two-tailed t-test). N.S indicates non-significant. Error bars represent mean ± SD. Source data are provided a Source Data file.

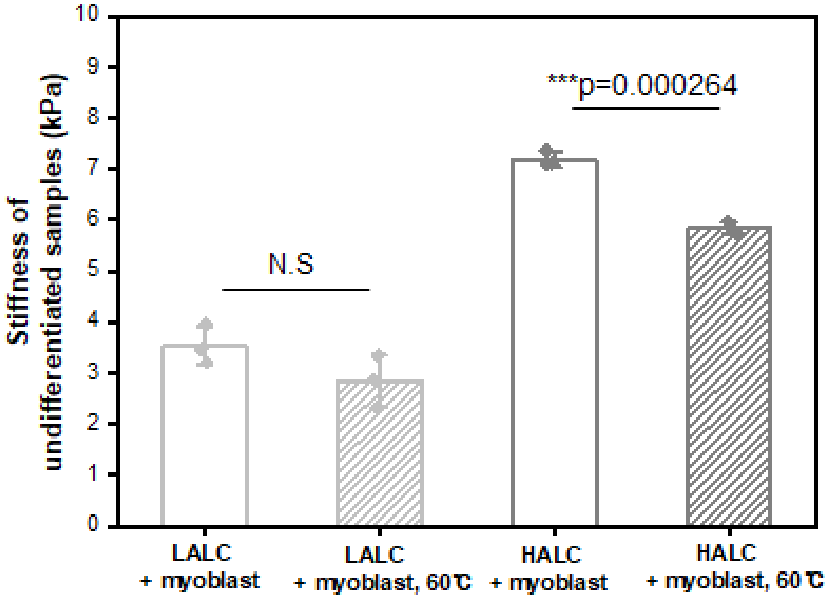

Supplementary Fig. 9: Change in the stiffness of the undifferentiated samples before and after cooking at 60 °C. The stiffness of the undifferentiated group was measured by frequency sweep at the angular frequency range from 0.1 to 1 (rad/s) (n = 3 independent experiments, two-tailed t-test). N.S indicates non-significant. Error bars represent mean ± SD. Source data are provided a Source Data file.

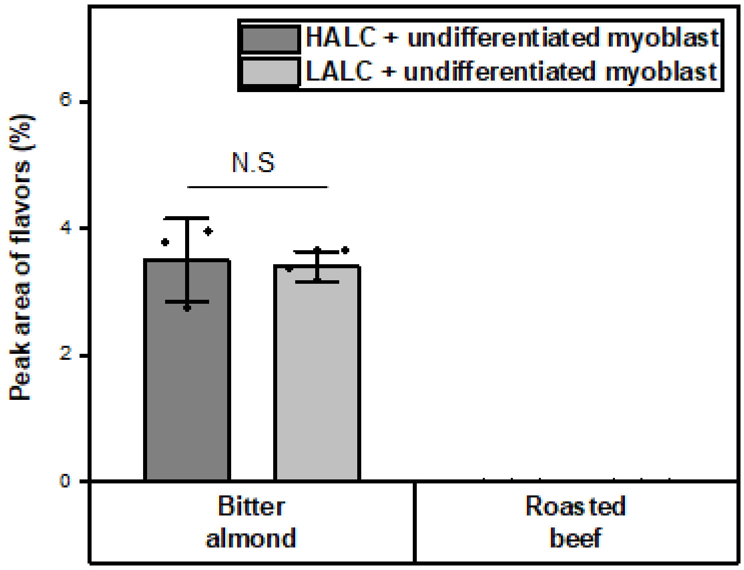

Supplementary Fig. 10: Flavor analysis of the undifferentiated myoblast samples performed using GC-MS. Peak area ratio of the flavor detected in the undifferentiated samples are shown (n = 3 independent experiments, two-tailed t-test). N.S indicates non-significant. Error bars represent mean ± SD. Source data are provided a Source Data file.

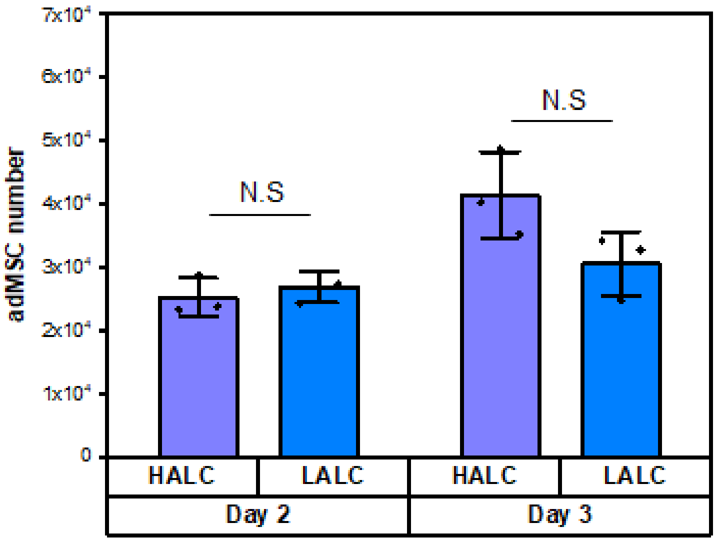

Supplementary Fig. 11: Evaluation of cell viability of the bovine adMSCs. Viability of the bovine adMSCs in each group was analyzed using CCK-8 assay. The cell numbers on proliferation day 2 and day 3 were compared (*n* = 3 independent experiments, two-tailed t-test). N.S indicates non-significant. Error bars represent mean ± SD. Source data are provided a Source Data file.

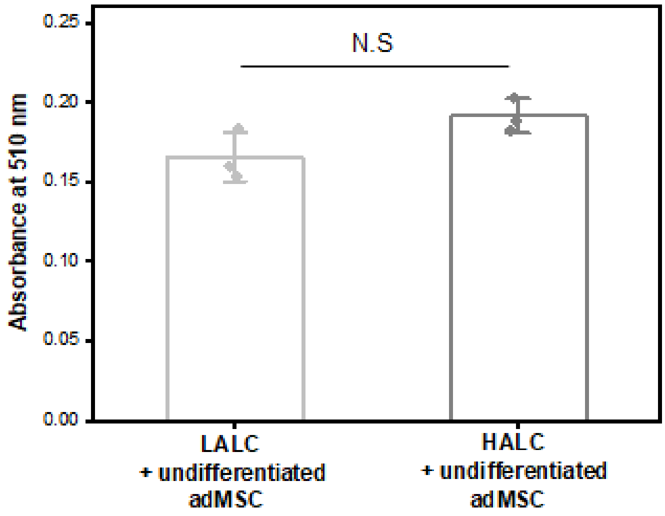

Supplementary Fig. 12: Adipogenic differentiation degrees of the undifferentiated samples. Quantification of the Oil Red O staining of the undifferentiated adMSC cultured on each scaffold was performed on the proliferation day 3 (n = 3 independent experiments, two-tailed t-test). N.S indicates non-significant. Error bars represent mean ± SD. Source data are provided a Source Data file.

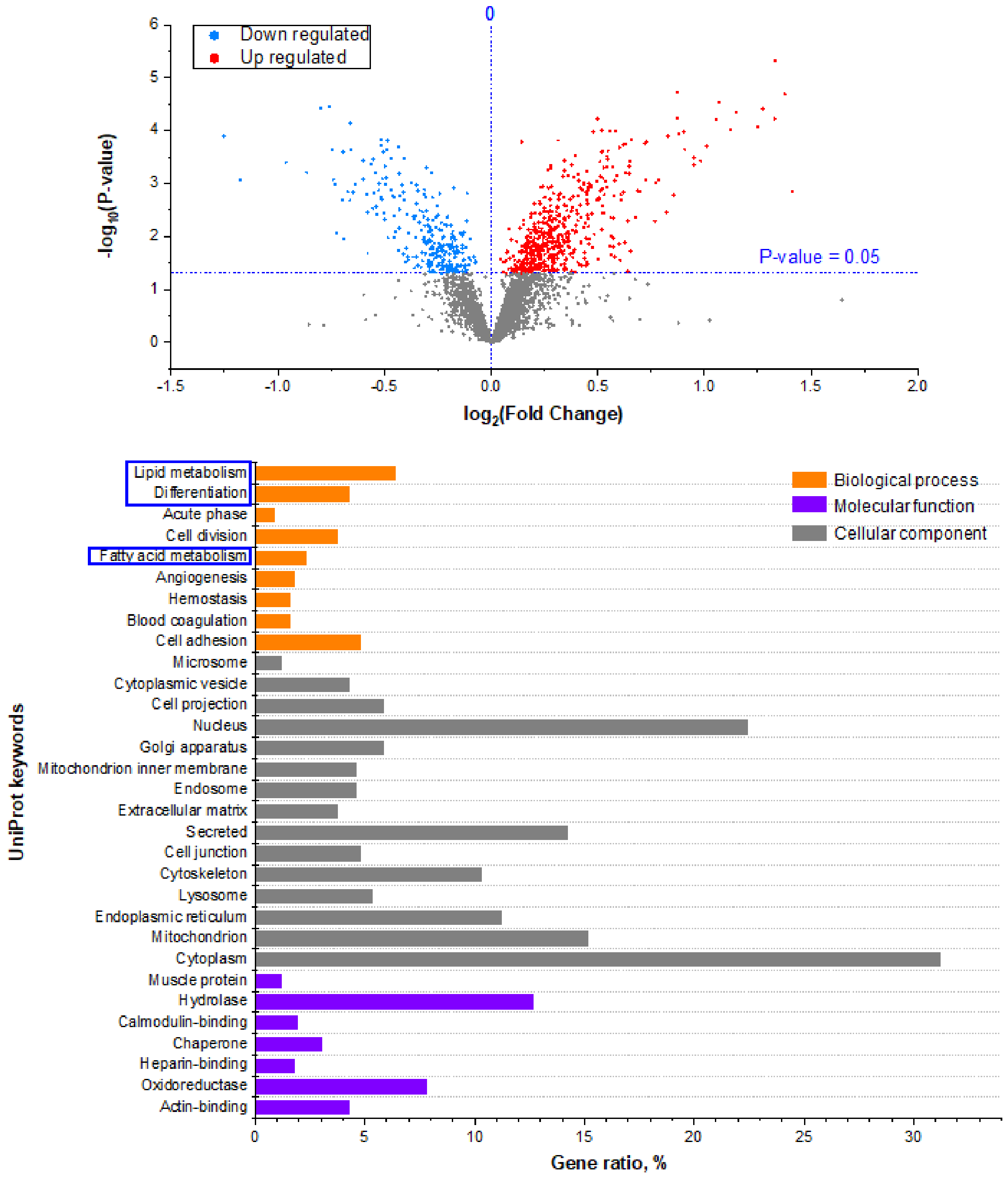

Supplementary Fig. 13: Proteins upregulated in the differentiated adMSCs in LALC. Volcano plot indicates the significantly down-regulated (blue dots) and up-regulated (red dots) proteins in LALC compared to that in HALC (n = 3 independent experiments, two-tailed t-test). Fold change indicates the ratio of the protein expression intensity in LALC to the protein expression intensity in HALC. Bar graph below the volcano plot shows the gene annotation of the proteins that are up-regulated in LALC. The X-axis indicates the proportion of the genes

with function corresponding to the gene annotation, whereas the Y-axis is the description of the gene functions. Source data are provided a Source Data file.

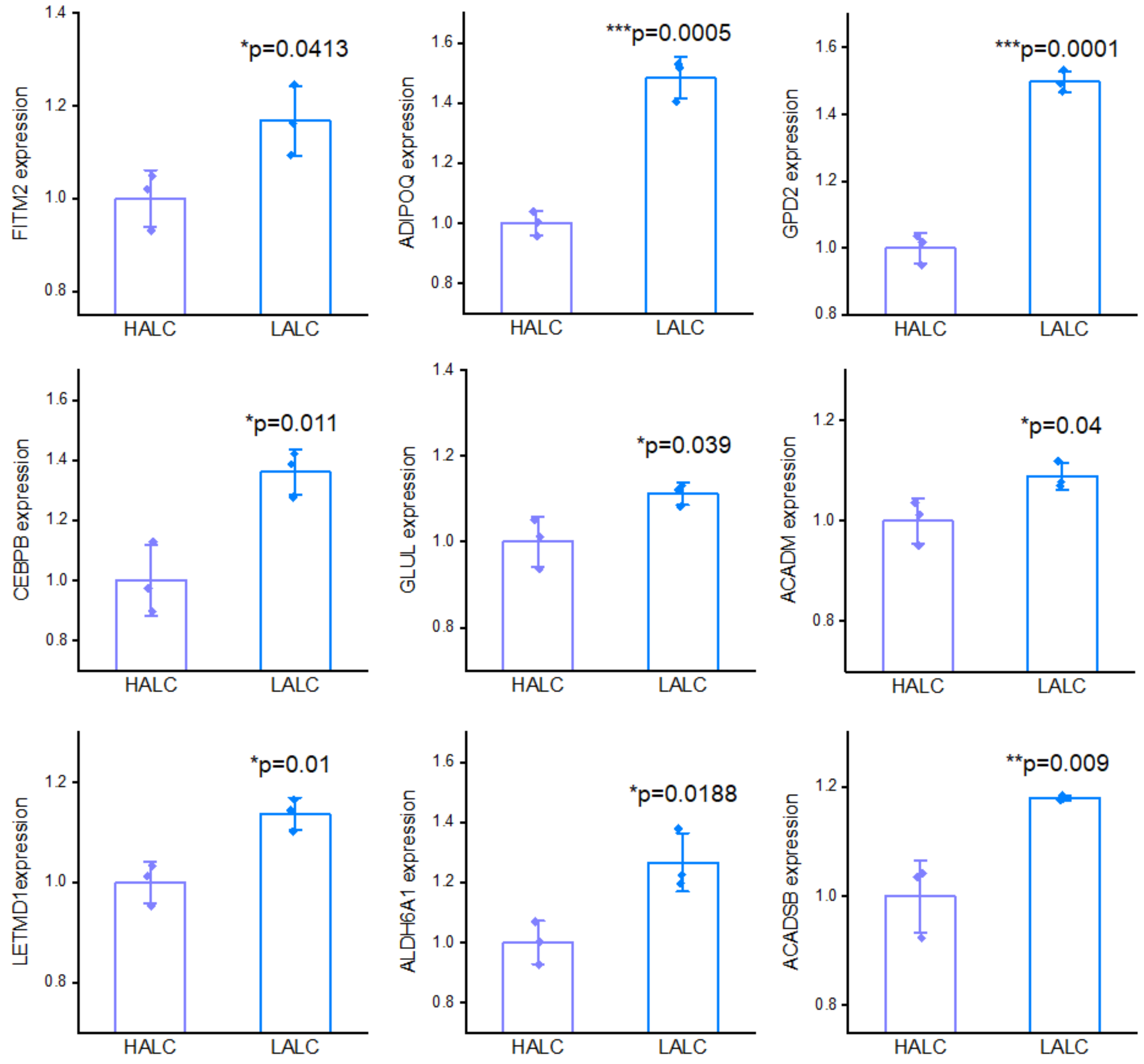

Supplementary Fig. 14: Proteins related to adipogenesis which are upregulated in the differentiated adMSCs in LALC. Expression of the adipogenesis-related proteins in the differentiated adMSCs in LALC were normalized to that of HALC (n = 3 independent experiments, two-tailed t-test). Error bars represent mean ± SD. Source data are provided a Source Data file.

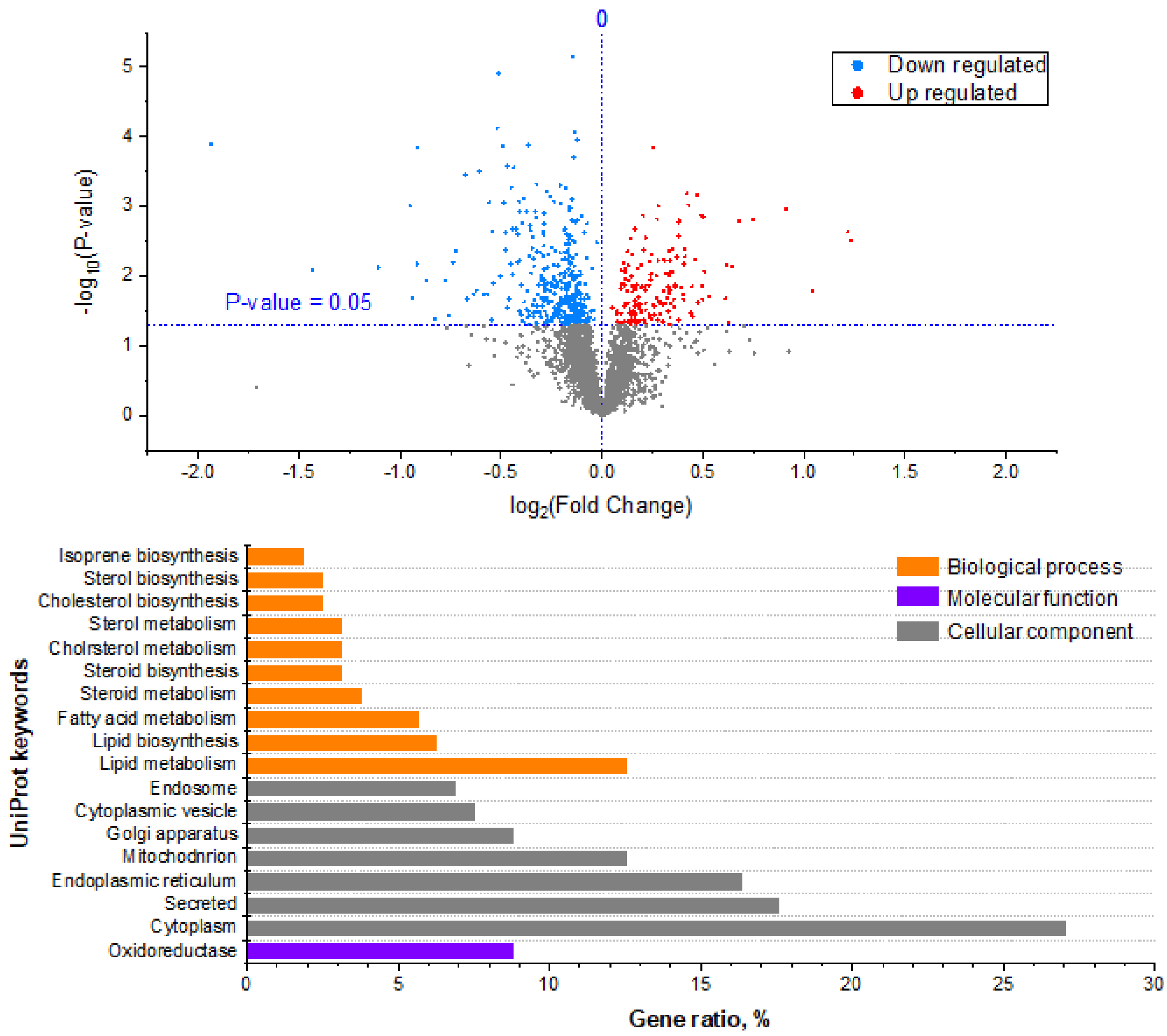

Supplementary Fig. 15: Proteins expressed in the undifferentiated adMSCs. Volcano plot indicates the significantly down-regulated (blue dots) and up-regulated (red dots) proteins in LALC compared to that in HALC (*n* = 3 independent experiments, two-tailed t-test). Fold change indicates the ratio of the protein expression intensity in LALC to the protein expression intensity in HALC. Gene annotation of the up-regulated in HALC is shown below the volcano plot. The X-axis indicates the proportion of the genes with function corresponding to the gene annotation, whereas the Y-axis is the description of the gene functions. Source data are provided a Source Data file.

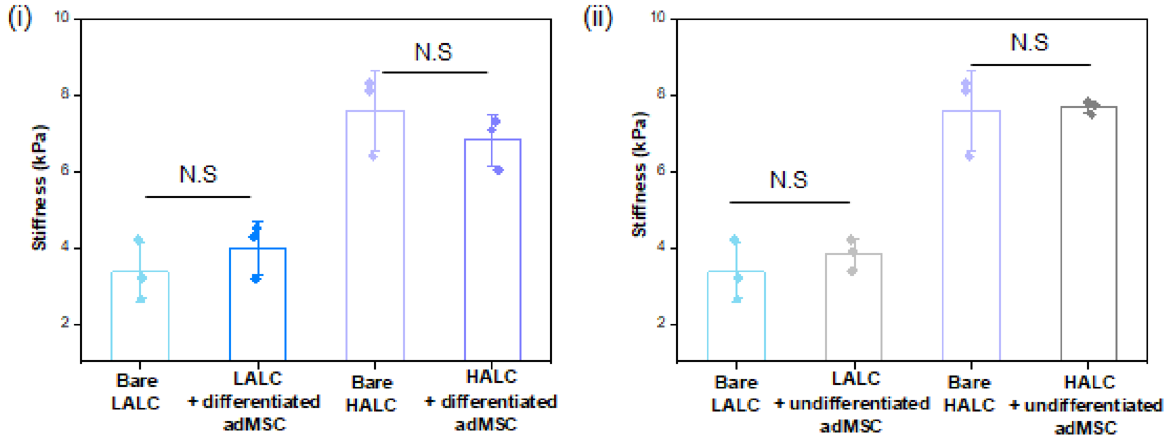

Supplementary Fig. 16: Evaluation of the stiffness change according to adipogenic differentiation. Stiffness of each group was measured by frequency sweep at the angular frequency range from 0.1 to 1 (rad/s) (n = 3 independent experiments, two-tailed t-test). N.S indicates non-significant. Error bars represent mean ± SD. Source data are provided a Source Data file.

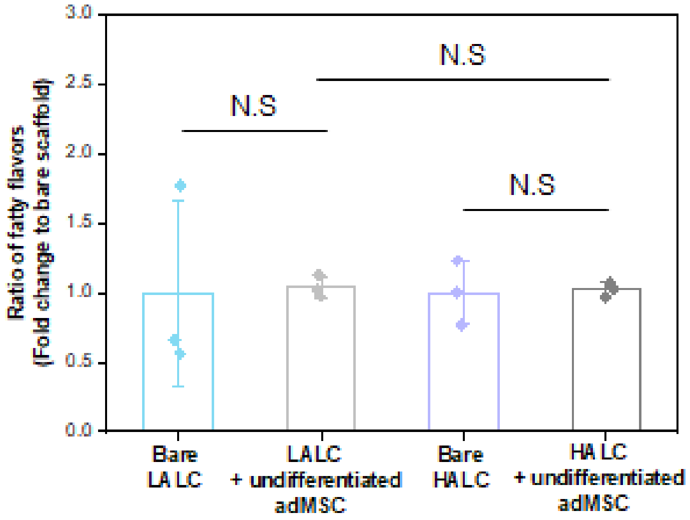

Supplementary Fig. 17: Flavor analysis of the undifferentiated groups. Fatty flavors detected in the undifferentiated groups were normalized to that of bare scaffolds (*n* = 3 independent experiments, two-tailed t-test). N.S indicates non-significant. Error bars represent mean ± SD. Source data are provided a Source Data file.

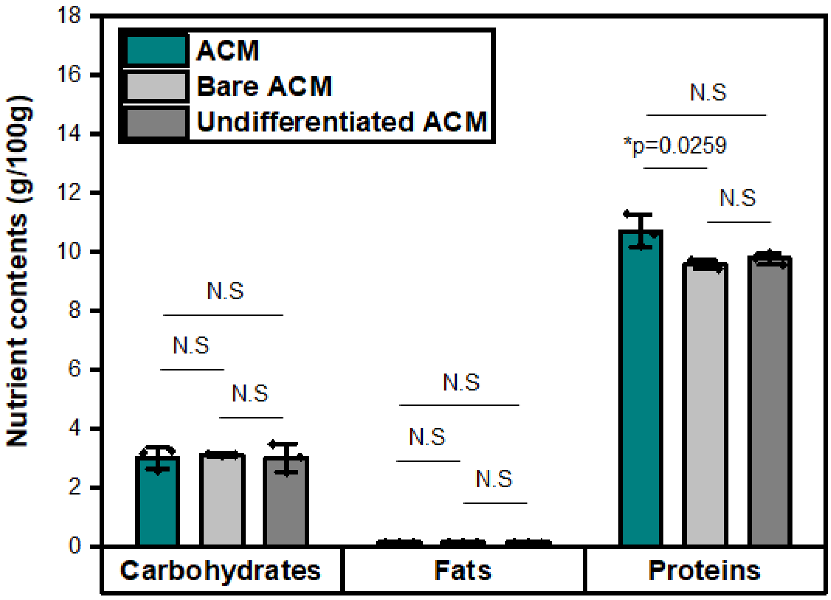

Supplementary Fig. 18: Nutrition factors of the ACM, bare ACM, and undifferentiated ACM. Nutritional analysis of carbohydrates, fats, and proteins was conducted for the three experimental groups (n = 3 independent experiments, two-tailed t-test). Fats include saturated and unsaturated fats. N.S indicates non-significant. Error bars represent mean ± SD. Source data are provided a Source Data file.

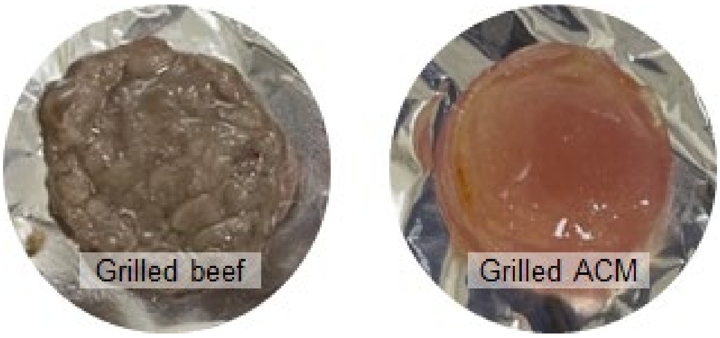

Supplementary Fig. 19: Images of the grilled beef brisket and grilled ACM. Beef brisket with the muscle:fat ratio of 3:1 was cut into the same dimension as that of the ACM. Then, both the samples were grilled with olive oil at 180 ℃. Browning, color change due to the Maillard reaction, was identified in both the samples.

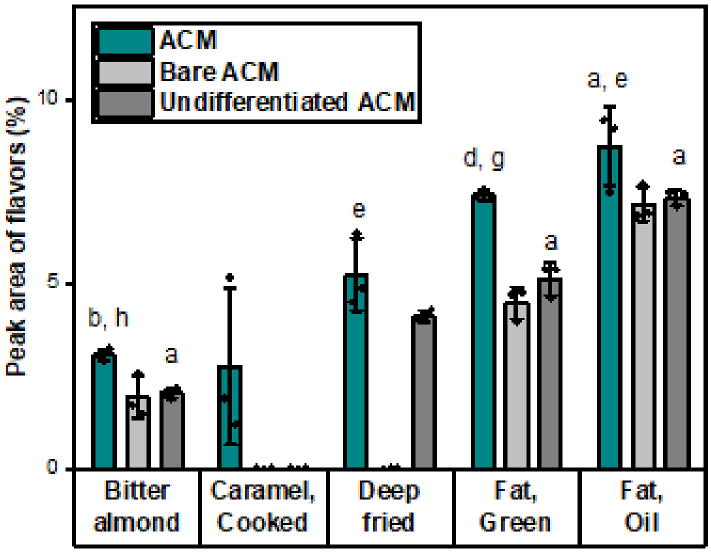

Supplementary Fig. 20: Comparison of the flavors detected in each group. Flavors were detected in ACM, bare ACM (assembled scaffolds without cells), and undifferentiated ACM (assembled scaffolds with undifferentiated cells) by GC-MS. Significancy was indicated as: a: non-significant (N.S) to bare ACM, b: $0.01 < \text{p-value} < 0.05$ to bare ACM, c: $0.001 < \text{p-value} < 0.01$ to bare ACM, d: $0.0001 < \text{p-value} < 0.001$ to bare ACM, e: N.S to undifferentiated ACM, f: $0.01 < \text{p-value} < 0.05$ to undifferentiated differentiated ACM, g: $0.001 < \text{p-value} < 0.01$ to undifferentiated ACM, h: $0.0001 < \text{p-value} < 0.001$ to undifferentiated ACM ($n = 3$ independent experiments, two-tailed t-test). Error bars represent mean ± SD. Source data are provided a Source Data file.

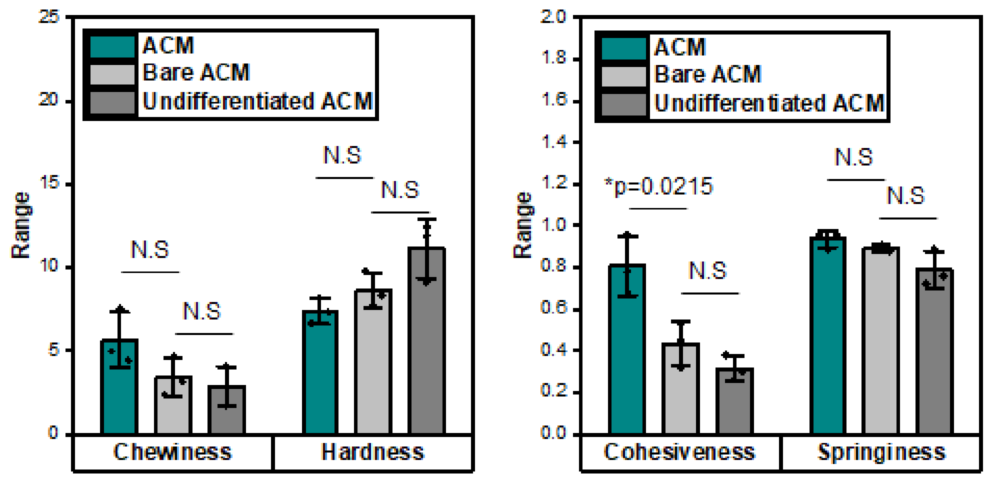

Supplementary Fig. 21: Results of the texture profile analysis of each group. Factors that determine the texture (chewiness, hardness, cohesiveness, springiness) were measured for ACM, bare ACM, and undifferentiated ACM after grilling the samples at 180 ℃ to confirm the effect of the cell differentiation quality control on the cultured meat texture (n = 3 independent experiments, two-tailed t-test). Error bars represent mean ± SD. Source data are provided a Source Data file.

Hydrogel	Gelatin concentration	Alginate concentration	Microbial transglutaminse concentration	$CaCl_2$ concentration
LALC	10% (w/v)	0.25% (w/v)	1% (w/v)	0.125% (w/v)
LAHC	10% (w/v)	0.25% (w/v)	1% (w/v)	0.25% (w/v)
HALC	10% (w/v)	2% (w/v)	1% (w/v)	1% (w/v)
HAHC	10% (w/v)	2% (w/v)	1% (w/v)	2% (w/v)

Supplementary Table 1. Composition of the hydrogels. Concentration of the polymers and crosslinkers in the hydrogels are shown in the table.

Hydrogel	Thickness (mm)
LALC	0.630±0.026458
HALC	0.633±0.020817

Supplementary Table 2. Thickness of the scaffolds. The thickness of each group was measured using a digital caliper (n = 3).

Category	Term	P-value	Gene ratio, %	Fold enrichment	Benjamini	FDR
Molecular function	Ribosomal protein	2.E-04	10.96	6.215	0.007	0.007
	Ribonucleoprotein	1.E-03	10.96	4.854	0.008	0.007
	Oxidoreductase	8.E-03	10.96	3.390	0.034	0.036
	Actin-binding	8.E-04	8.22	7.990	0.008	0.007
	Chaperone	7.E-03	6.85	6.369	0.034	0.036
	Muscle protein	8.E-04	5.48	21.587	0.008	0.007
Cellular component	Cytoplasm	8.E-23	64.38	4.229	2.05E-21	1.82E-21
	Nucleus	9.E-04	31.51	2.0	0.006	0.005
	Cytoskeleton	2.E-07	20.55	5.706	2.46E-06	2.18E-06
	Mitochondrion	7.E-04	16.44	3.337	0.006	0.005
Biological process	Glycolysis	7.E-04	5.48	21.561	0.035	0.035

Supplementary Table 3. Genes categorized according to the DAVID gene annotation. The genes related to the proteins which were expressed more than 1.5 folds in HALC than in LALC were clustered according to the DAVID gene annotation. (n = 3 independent experiments, two-tailed t-test), Source data are provided a Source Data file.

Cas Number	Compound Name	Flavors
123-32-0	2,5-Dimethylpyrazine	Roasted beef
100-52-7	Benzaldehyde	Bitter almond

Supplementary Table 4. List of the flavor compounds detected from the differentiated myoblasts.

Cas Number	Compound Name	Flavors
124-19-6	Nonanal	Fat, floral, green
104-76-7	2-ethyl-1-hexanol	Fatty, green

Supplementary Table 5. Flavor compound detected from the adMSC groups.

Cas Number	Compound Name	Flavors
66-25-1	Hexanal	Fat, oil
124-19-6	Nonanal	Fat, green
111-87-5	Octanol	Bitter almond
98-00-0	Furan-2-ylmethanol	Burnt, caramel, cooked
25152-84-5	Decadienal	Deep fried
110-62-3	Valeric aldehyde	Almond, malt
590-86-3	3-methylbutyraldehyde	Fatty, almond
513-86-0	Acetoin	Butter, Creamy

Supplementary Table 6. List of the flavor compounds detected from the grilled ACM and grilled beef.

Reagent name	Company	Catalog number
Alginic acid sodium salt	Sigma-Aldrich	180947
Calcium chloride	Sigma-Aldrich	C5670
70% ethanol	DAEJUNG	4018-4410
Antibiotic-antimyocotic solution	Welgene	LS203-01
Dubecco's phosphate-buffered saline	Welgene	LB001-02
High glucose-Dulbecco's modified eagle medium	Welgene	LB001-05
Heat-inactivated fetal bovine serum	Welgene	S101-01
Red blood cell lysis buffer	Sigma-Aldrich	11814389001
basic fibroblast growth factor	Peprotech	100-18B
Trypsin-EDTA	Welgene	LS015-10
Low-glucose Dulbecco's modified Eagle's medium	Welgene	LM001-01
Phosphate buffered saline	Gibco® Life Technologies	10010031
Horse serum	Gibco® Life Technologies	26050088
Penicillin-streptomycin-glutamine	Gibco® Life Technologies	10378016
Insulin from bovine pancreas	Sigma-Aldrich	I6634
Dexamethasone	Sigma-Aldrich	D4902
Ciglitizone	Sigma-Aldrich	C3974
Oleic acid	Sigma-Aldrich	O1383
Cell Counting Kit-8	Dongin LS, Korea	CCK-3000
Bovine serum albumin	Sigma-Aldrich	A3311
Triton™ X-100 solution	Sigma-Aldrich	X100
MF20	DSHB	AB2147781
Donkey anti-mouse Alexa flour 594	Thermo Fisher	A21203

DAPI	Sigma-Aldrich	D9542
HCS LipidTOX™ Red Neutral Lipid Stain	Invitrogen	H34476
RIPA Lysis and Extraction Buffer	Thermo Fisher	89900
Pierce™ BCA Protein Assay Kit	Thermo Fisher	23227
MYH1 ELISA	MyBioSource	MBS7229767
TMTpro™ 16plex Label Reagent Set	Thermo Fisher	A44520
Fish gelatin	GELTECH	Fish gelatin 150 ~ 250 Bloom
Microbial transglutaminase (ACTIVA TG-B)	AJINOMOTO	ACTIVA TG-B
Ethanol	Samchun Chemical	E0220
Collagenase, type 2	Worthington Biochemical Corporation	LS004174
Pronase	Calbiochem	53702
Sodium dodecyl sulfate	Sigma-Aldrich	L3771
Iodoacetamide	Sigma-Aldrich	I1149
Phosphoric acid	Sigma-Aldrich	93752
Trypsin Gold	Promega	V5280
Tetraethylammonium bromide	Thermo Fisher	90114
Methanol	Supleco	1060074000
Formic acid	Fisher chemical	A117-50
Acetonitrile	J.T Baker	9017-88
Sodium hydroxide solution	Sigma-Aldrich	72068
Hydrochloric acid	DAEJUNG	1005-4100

Supplementary Table 7. List of the reagents used in the method section.
